# Supplementary material for: Effect of an extension speech training program based on Chinese idioms in patients with post-stroke non-fluent aphasia: A randomized controlled trial
Source: PLoS One. 2023 Feb 8;18(2):e0281335. doi: 10.1371/journal.pone.0281335 (PMC9907817; doi:10.1371/journal.pone.0281335)
Supplement: S1 Protocol — (PDF) [file pone.0281335.s003.pdf]

项　目　名　称：计算机辅助非流畅性失语症成语朗读康复训练

项目负责人姓名：何小俊

联 系 方 式：13908653137

科　　　　室：干部保健处

项 目 联 系 人：李薇薇

联 系 方 式：15927369892

研 究 单 位 名 称：武汉大学人民医院

方 案 版 本 号：1.0

日　　　　期：2020 年 4 月 13 日

研究方案摘要

项目名称：计算机辅助非流畅性失语症成语朗康复训练

研究目的：探讨成语朗读对非流畅性失语症的康复护理效果，以期能寻求一种新的失语症康复训练有效手段，来满足非流畅性失语症患者的康复需求。

设计类型：随机对照研究

研究对象：脑卒中后非流畅性失语症患者

样本量：基于本研究的方法和目的进行预试验，参照预试验结果，使用公式 n=(μα+μβ) 2/[2 (sin-1Ｐe-sin-1Ｐc) 2]计算样本量，Pe、Pc 分别为试验组和对照的阳性率（即语言功能有所改善的患者占比率） 。

入排标准

纳入标准：

（1）通过中国康复研究中心《失语症筛查表》检查被诊断为失语症，并且为非流畅性失语症患者；

（2）小学或以上文化程度；

（3）意识清楚能合作，发病前智力正常，无精神疾病史；

（4）为缺血性脑卒中后非流畅性失语症，脑梗部位为优势侧额下回后部， 疾病处稳定期，能坚持独坐 30min 以上；

（5）知情并同意参加本次测试，签署知情同意书；

（6）年龄在 45~70 岁

排除标准：

（1）认知障碍；

（2）精神障碍；

（3）双眼矫正视力＜1.0 或视野缺损；

（4）听力障碍；

（5）构音障碍；

（6）完全性失语、Wernicke 失语、经皮质感觉性失语及经皮质混合性失语；

（7）言语失用；

基线资料

性别、年龄、文化程度、脑卒中类型、病程、脑梗面积、汉语失语检查法（ABC）及日常生活交流能力评定法（CADL）各项评分；

观察指标

汉语失语检查法(ABC)各项目评分，包括听理解、口语表达、书写、阅读；日常生活交流能力评定法（CADL）各项指标，包括22项日常交流活动；

统计分析方法

两独立样本 t 检验，配对样本 t 检验

3. 研究方案正文

3.1 研究背景和立项依据

失语症是一种脑损害（一般在左侧大脑）后交流能力损害或丧失的获得性语言障碍，即是指对理解和形成语言符号能力受损，对语言成分解码和编码能力受损[1]的语言障碍，脑卒中后至少有 1/3 的患者出现失语症[2-3]。其主要临床特征表现有：口语产生障碍、听理解障碍、复述障碍、命名障碍、阅读（朗读和理解）障碍和书写障碍等。根据失语症的主要临床特点，失语症分为：Broca（运动性）失语、Wernicke（理解性）失语、传导性失语、经皮质运动性失语、经皮质感觉性失语、经皮质混合性失语、完全性失语和命名性失语[4]。而根据患者的口语将失语症又分为为流畅性失语和非流畅性失语两大类[1]。非流畅性失语症常见临床表现是语量异常稀少，常常每分钟少于 50 个字，甚至每分钟不到 10 个字，说话费力是其另一个特征；流畅性失语症特点是说话量多、说话不费力、发音和语调正常，但说话含信息量少[5-6]。尽管失语症被定义为语言障碍，但情感和社会心理的改变常常伴随失语症出现[7]。据报导，失语症被认为是卒中后出现情感抑郁、社会孤立和生活质量低下的重要因素[8]。与他人语言交流是形成社会人际关系的第一步[9],大多数临床专家认为的，有效的交流是保证失语症患者生活质量不可缺少的一部分[9]。失语使患者的生活质量受到显著影响，因此失语症语言康复护理应受到临床康复护理人员高度重视。

国内外失语症的康复训练方法有很多，包括例如⑴Schuell 刺激疗法，是指以对损害的语言符号系统应用有控制的、强的听觉进行刺激为基础，尽最大可能的促进患者的语言功能重建和交流能力恢复，是多种失语症治疗方法的基础，是应用最广泛的语言治疗方法之一[5]。⑵强制诱导性语言治疗(CILT)，其采用强制口语交流和抑制非口语交流形式进行大量有目标的语言训练。与传统

语言治疗相比，CILT 的优势在于它能短时间内提高语言功能，成功地将改善的功能应用于日常生活[10]；⑶经颅磁刺激（TMS）,是通过在头皮上放置一个“8”字形绝缘铜线圈，利用磁场在离散性大脑区域形成电流，使之产生一个快速波动的磁场穿透头骨而使大脑皮质神经元去极化，以达到促进语言功能的恢复效果[11]；⑷针刺激，指采用中国古典经穴针刺的方法，中医经络学说认为不同穴位有其特殊功效，现代医学通过功能影像学试验显示不同穴位具有不同特殊脑激活区域，而这些脑激活区域也许正和针刺的疗效相对应[12]；（5）旋律语调治疗法（MIT)，利用语言当中音调、韵律、重音、节奏等旋律特点来促进患者言语功能恢复[13]等。目前，脑卒中失语症患者语言障碍的康复治疗复杂多样,然而因失语症患者失语类型、严重程度、性别、民族、文化、年龄、爱好等的不同，对失语症有效康复护理措施需求也不一。因此，为满足不同失语症患者的康复需求，最大的提高语言康复护理效果，康复护理人员需要不断的寻求更多新的失语症康复训练有效手段。

旋律语调治疗(Melodic Intonation Therapy,MIT) 从 20 世纪 70 年代逐渐发展起来并沿用至今，通过利用语言当中音调、韵律、重音、节奏等旋律特点来促进患者言语产出，它是一种结构化的失语症治疗程序[13]。MIT 作为非流畅性失语症的治疗方法，其效果是众多训练方法中少数得到公认的[14]。并且对于听理解良好但语言表达受损的这类失语症患者，MIT 的治疗效果表现最为显著[15]。利用语言材料的特定旋律、语调、节奏、重音和左手叩击来提高患者的口语产出是 MIT 的康复原理[16]。成语是汉语特有的词语搭配，虽然其旋律方面稍弱，但是它具有歌曲所不具备的特点：①成语搭配固定工整，由 4 字组成，有较好的节奏感；②具有一定的故事背景，有利于患者词语的提取和口语表达的延伸，并且成语故事的背景特性，使训练更加具有趣味性；③具有广泛的大众熟悉度[16,17]。本研究受 MIT 的启发，利用中国汉语成语这些特性，为非流畅性失语症患者进行成语朗读训练，以期能改善患者语言功能。　　计算机作为信息化的主要工具，以其快速、便捷、形象等特点，在全世界普及。同时，随着“互联网+医疗”模式的发展，计算机在医疗领域发挥着巨大作用，其图文、声像、动画等多媒体变化特点，为失语症患者康复提供了较大强度的技术支持，因此，计算机被认为是一种有用的语言康复手段[18]。计算机辅助失语症语言评

估和计算机辅助失语症康复治疗是计算机在失语症康复护理中应用的两个主要方面。计算机辅助失语症语言评估是指将纸质版的失语症评估量表图片和文字转化在电脑上，后通过屏幕和语音呈现，从而对失语症患者进行语言评估。国内外出现的计算机辅助失语症语言功能评估软件主要包括：语言障碍诊疗仪 ZM2 .1 [19]、计算机言语评估及康复系统 TG PX 111[20]和 Parrot 系列失语症治疗[21]等。计算机辅助失语症康复护理主要包括三种形式[22]：第一种是通过语义、音韵或语法的特质来实现受损语言功能恢复，即障碍导向治疗法；第二种是强调实现患者日常生活交流活动，训练患者尽可能有效使用残存语言的功能治疗；第三种是通过使用计算机鼓励患者多参与社会交流的参与式治疗，其中当前大多数失语症康复计算机软件都倾向于障碍导向治疗。国内常见的失语症康复护理计算机软件有语言障碍诊疗仪 ZM2.1[19]、无障碍电脑语言系统 U1[23]和计算机言语评估及康复系统 TG PX 111[20]等；国外计算机软件见于计算机脚本训练 Aphasia Scripts™[24]、IMITATE 计算机辅助失语症治疗系统[25]和 Parrot 系列失语症治疗软件[21]等。Katz 认为，计算机在失语症康复中的应用主要分为两种，一种是计算机辅助治疗（CAT），另一种是计算机独立治疗（COT）[26]。

3.2 研究目的

主要目的：探讨成语朗读对非流畅性失语症的康复护理效果，以期能寻求一种新的失语症康复训练有效手段，来满足非流畅性失语症患者的康复需求。

3.3 研究设计

本研究为随机对照研究。在研究过程中，不会对受试者进行或提供任何随机或任何以研究方案驱动的治疗。如果在临床上适用，则由治疗医师酌情做出治疗决定以及选择治疗方案。

3.3.1 受试人群

本研究的受试人群是脑卒中后非流畅性失语症患者。

入选标准：

（1）通过中国康复研究中心《失语症筛查表》检查被诊断为失语症，并且为非流畅性失语症患者；

（2）小学或以上文化程度；

（3）意识清楚能合作，发病前智力正常，无精神疾病史；

（4）为缺血性脑卒中后非流畅性失语症，脑梗部位为优势侧额下回后部， 疾病

处稳定期，能坚持独坐 30min 以上；

（5）知情并同意参加本次测试，签署知情同意书；

（6）年龄在 45~70 岁

排除标准：（指即使符合入选标准但是还不符合研究想要的对象）

(1) 认知障碍；

(2) 精神障碍；

(3) 双眼矫正视力＜1.0 或视野缺损；

(4) 听力障碍；

(5) 构音障碍；言语失用；

(6) 完全性失语、Wernicke 失语、经皮质感觉性失语及经皮质混合性失语；

3.3.2 研究所需样本量的确定

基于本研究的方法和目的进行预试验，参照预试验结果，使用公式 n=(μα+μβ) 2/[2 (sin-1Ｐe-sin-1Ｐc) 2]计算样本量，Pe、Pc 分别为试验组和对照的阳性率（即语言功能有所改善的患者占比率）。

3.3.3 研究方法及步骤

由于这是一项随机对照研究，医生将根据说明书和临床路径决定治疗计划。研究者将会查看患者的病史和检验报告，根据入选和排除标准确定患者的资格。患者必须在执行数据收集之前,签署最新的经 IRB/IEC 批准的知情同意书(ICF)。在这项非干预研究中，研究者在基线及干预后（2 周）收集临床评估数据。

（1）知情同意及入组

提供了知情同意书，并符合所有其他入选/排除标准的受试者，被视为入组本研究。

（2）受试者识别号

通过随机数字表法进行分组，每位受试者给予独特的识别号（由一位研究者按照随机数字表法选择第三行第二列依次选取数字，所有数字除以 2，奇数分到对照组，偶数分到观察组）。所有研究文件(如语言评估表等)将采用该识别号。此外，根据数据隐私法规，允许使用独特的识别号，只要其不含有能识别受试者身份的组合信息。

（3）研究过程：

观察组在接受常规治疗（包括药物治疗，神经内科常规护理，言语康复的宣

教指导等）的基础上予以计算机辅助失语症成语朗读训练，其具体方法为利用计算机软件 Powerpoint2003，计算机屏幕上依次呈现白底黑字的成语(成语来源于《学生实用成语词典》)，共５０个，电脑屏幕会依次显示成语，成语以文字的方式出现,成语均配有辅助朗读的提示材料，包括相应成语拆封或扩展成字、词、句、段落的形式。在屏幕下方依次有各个提示的按键和切换下一个成语的按键，首先是言语康复师请患者朗读成语，当患者不能朗读成语时，则按需点击相应的提示按键（语音、视频或是图片提示），以帮助患者朗读。训练周期为2周，每天 40 分钟，训练场地为临床教室。

对照组仅进行常规治疗，包括药物治疗，神经内科常规护理，言语康复的宣教指导，但不进行规律的言语康复训练。于研究前、后，采用汉语失语检查法（ABC）及日常生活交流能力评定法（CADL）量表评估其语言功能。

（4）数据来源/数据收集过程

通过提取入组受试者常规临床诊疗记录获取这项研究的数据。要求研究者在整个监测期间根据患者病历中输入的信息填写研究的电子病例报告表(eCRF)和或记录表中。

（5）数据收集步骤：（根据研究内容自行填写）

基线期：一般信息表和ABC及CADL量表评分

常规诊疗期：ABC及CADL量表评分

3.4 数据管理

把研究对象的数据迅速、完整、无误地纳入报告，所有涉及数据管理的各种步骤均需记录在案，以便对数据质量及试验实施进行检查。保证数据库的保密性，应具有计算机数据库的维护和支持程序。试验前需设计可被计算机阅读和输入的临床报告表及相应的计算机程序。研究对象分配必须按试验设计确定的随机方案进行，每名研究对象的密封代码应由研究者保存。

原始资料记录的制度：1) 在研究开始前，项目负责人应与研究人员、监察员讨论如何在原始资料中记录有关临床研究的信息，并建立对原始记录的要求。2) 项目负责人提供原始资料中临床研究信息记录的格式（CRF 表）。3) 原始资料按医疗文件的行业惯例由完成的研究人员签字并注明日期。4)在资料的每一页上均应有研究对象的姓名和研究编号。 5) 所有原始资料的更正应由进行

更正的研究者签名并注明日期。6)所有在病例报告表上记录的信息和数据，均应出自原始资料中的记录。7)病例报告表的填写与移交：CRF 填写是在患者入组后由项目指定专人按照患者病例号找到相应的电子或纸质病历如实填写患者入组流水号并录入 CRF 表，在填写完成后由参加纳入患者的研究者审核后签名。 8)项目知情同意书在患者签署后保存在每一份电子 CRF 表中。9) 填写完成的病例报告表，在干预及早期随访完成后将送交参加本临床试验的数据管理人员统一建立数据库并由项目指定录入人员双份录入，由研究者保存锁定和保存电子版原始数据，后期随访数据在结束随访后仍交由项目指 定录入人员双份录入。10) 数据的录入与修改：在各阶段录入的由参加纳入患者的研究者审核签名、监察员签字后将锁定所录入的该阶段的相关病历信息。所有数据将采用计算机软件编制数据录人程序进行双份录入。在此期间，将有疑问的 CRF 表通过临床监察员转交研究者进行数据审核，研究者应尽快回答并返回。11) 数据锁定： 锁定后的数据文件不允许再作变动。数据库将交统计分析人员按统计计划书要求进行统计分析。

3.5 统计分析

3.5.1 统计方法

采用统计学软件 SPSS20.0 进行统计学分析，数据用（ $\bar{x}$±s）表示，本研究语言功能评分和患者一般资料（年龄和病程）为计量资料，因此实验组和对照组两组间比较采用两独立样本 t 检验，两组各自内部前后评分比较采用配对样本 t 检验，研究前后两组语言功能变化差值均数的比较采用配对样本 t 检验；另外患者一般资料（包括性别和卒中类型）为计数资料，采用卡方检验，显著性水平 α =0.05。

3.5.2 统计软件

SPSS20.0

3.5.3 研究质量管理

（1） 本研究负责人具有正高职称，主持并完成多项课题，发表数篇跟老年患者康复有关的论文，具备完成此研究的研究基础。

（2） 本研究组成人员有康复科主任医师朱珊珊和康复科医师鲁银山，具有多年语言康复训练的经验、可以为研究提供训练工具和场地，此外，研究组成

人员有老年病科护师李薇薇（研究生）和两名老年护理专业的研究生，具有老年康复相关方面的经验和知识，能胜任研究的具执行。

3.6 安全性评价

本研究干预措施为语言功能康复训练，干预措施中无侵入性操作和药物治疗，在对患者实施干预前和干预的过程中，均有本科室医生对患者进行病情评估，确定是否适合进行和继续参加语言康复训练。在干预过程中患者有任何不适，均可示意医护人员。此外，本研究干预均在患者所在科室内进行，无需外出。

3.7 研究预期进度和完成日期

2020 年 9 月 13 日-2021 年 9 月 17 日

项目负责人签字：

2020 年 4 月 10 日
