# Supplementary material for: Effect of an extension speech training program based on Chinese idioms in patients with post-stroke non-fluent aphasia: A randomized controlled trial
Source: PLoS One. 2023 Feb 8;18(2):e0281335. doi: 10.1371/journal.pone.0281335 (PMC9907817; doi:10.1371/journal.pone.0281335)
Supplement: S2 Protocol — (PDF) [file pone.0281335.s004.pdf]

Project name: Computer-assisted non-fluent aphasia idiom reading rehabilitation training

Name of the project leader: He Xiaojun

Contact information: 13908653137

Department: Cadre Health care Department

Project contact person: Li Weiwei

Contact information: 15927369892

Name of the research unit: The People's Hospital of Wuhan University

Scheme version number: 1.0

Date: April 13,2020

Summary of the study protocol

Project name: Computer-assisted non-fluent aphasia idiom Lang rehabilitation training

Objective: To explore the effect of idiom training on non-fluent aphasia, in order to find a new effective means of aphasia rehabilitation training to meet the rehabilitation needs of non-fluent aphasia patients.

Design type: a randomized controlled study

Study jects: Non-fluent aphasia patients after stroke

Sample size: Based on the method and purpose of this study, according to the results of formula n= (+) 2 / [2 (sin-1 e-sin-1 c) 2], Pe and Pc are the positive rates of the test group and the control respectively (i. e., the proportion of patients with improved language function).

Entry standard

Inclusion criteria:

(1) Was diagnosed with aphasia through the aphasia Screening Form of China Rehabilitation Research Center, and was a non-fluent aphasia patient;

(2) Primary school education or above;

(3) Clear awareness and cooperation, normal intelligence before the onset, and no history of mental illness;

(4) For non-fluent aphasia after ischemic stroke, the cerebral infarction site is the posterior inferior frontal gyrus of the superior side, and the disease is stable, and you can sit alone for more than 30min;

(5) Informed and agreed to participate in this test, and sign the informed consent form;

(6) Age is from 45 to 70 years old

Exclusion criteria:

(1) Cognitive impairment;

(2) Mental disorders;

(3) Binocular corrected visual acuity <1.0 or visual field defect;

(4) Hearing impairment;

(5) Ararthria;

(6) Complete aphasia, Wernicke aphasia, transcortex sensory aphasia and transcortex mixed aphasia;

(7) Misuse of speech;

Baseline data

Gender, age, education, type of stroke, area of disease, cerebral infarction, Aphasia Battery Of Chinese(ABC)score, Comprehensive activities of daily living(CADL) scale score

Observational indicators

Aphasia Battery Of Chinese (ABC) scale,all items score, including listening to understanding, oral expression, writing,reading four items; Comprehensive activities of daily living(CADL) scale score,, including 22 daily communication activities

statistical analysis technique

Two independent sample t-test, paired sample t-test

3. Text of the study protocol

3.1 Research background and project approval basis

Aphasia is an acquired language disorder with impairment or loss of communication ability after brain damage (generally in the left brain), that is, impaired ability to understand and form language symbols, and impaired decoding and coding of language components[1]At least 1 / 3 of the patients developed aphasia after stroke[2-3]. Its main clinical characteristics are: oral English disorders, listening and comprehension disorders, retelling disorders, naming disorders, reading (reading and comprehension) disorders and writing disorders.According to the main clinical characteristics of aphasia, aphasia is divided into: Broca (Motor) aphasia, Wernicke (comprehension) aphasia, conductive aphasia, transcortical motor aphasia, transcortical sensory aphasia, transcortical mixed aphasia, complete aphasia, and named aphasia[4]. According to the patient's oral English, aphasia is divided into fluent aphasia and non-fluent aphasia[1]. The common clinical manifestation of non-fluent aphasia is unusually rare, often less than 50 words per minute, or even less than 10 words per minute. Hard speech is another feature; fluency aphasia is characterized by more speech, hard speech, normal pronunciation and intonation, but

less information[5-6]. Although aphasia is defined as a language disorder, emotional and psychosocial changes often accompany aphasia[7]. Aphasia is reported to be an important factor in emotional depression, social isolation and low quality of life after stroke[8]. Language communication with others is the first step in forming social interpersonal relations[9], Most clinical experts believe that effective communication is an indispensable part of ensuring the quality of life of patients with aphasia[9]. aphasia significantly affects the quality of life of patients, so language rehabilitation care for aphasia should be highly valued by clinical rehabilitation nursing staff.

There are many rehabilitation training methods of aphasia at home and abroad, including for example, ⑴Schuell stimulation therapy, refers to the damage of language symbol system application controlled, strong auditory stimulation, to promote the patients' language function reconstruction and communication ability recovery, is the basis of a variety of aphasia treatment, is one of the most widely used language therapy[5]. ⑵ uses compulsory induced language therapy (C ILT), which uses forced verbal communication and suppresses non-oral communication, and performs a lot of targeted language training.With the traditional

The advantage of CIL T over language therapy is that it can improve language function in a short period of time and successfully apply the improved function to daily life[10]; ⑶ transcranial magnetic stimulation (TMS) is performed by placing a ″figure 8″ insulated copper coil on the scalp, using the magnetic field to form a current in discrete brain areas, creating a rapidly fluctuating magnetic field through the skull, to achieve the recovery of language function[11]; ⑷ needle stimulation refers to the method of Chinese classical acupoint acupuncture. Traditional Chinese medicine meridian theory believes that different acupoints have their special effects. Modern medicine shows that different acupoints have different special brain activation areas through functional imaging tests, and these brain activation areas may be corresponding to the curative effect of acupuncture[12]; (5) Melodic intonation therapy (MIT), using the melodic characteristics such as tone, rhythm, stress, rhythm, to promote the recovery of patients’ speech function[13]class.At present, the rehabilitation treatment of language disorders in stroke aphasia patients is complex and diverse. However, due to the different types, severity, gender, nationality, culture, age, hobby, etc. of aphasia patients, the needs for effective rehabilitation care measures for aphasia are also different.Therefore, in order to meet the rehabilitation needs of different aphasia patients and maximize the improvement of language rehabilitation nursing effect, rehabilitation nursing staff need to constantly seek more new effective means of aphasia rehabilitation training.

Melodic Intonation Therapy (MIT) has gradually developed since the 1970s and is used today. It is a structured treatment procedure of aphasia by using melodic characteristics in language to promote

patient speech output. It is a structured procedure[13]. As the treatment of nonfluent aphasia, MIT is recognized by the few of many training methods[14]. And for such aphasia patients with good hearing comprehension but impaired speech expression, MIT is the most significantly effective in the treatment[15]. Using the specific melody, intonation, rhythm, stress, and left-hand percussion of the language material to improve the oral output of patients is the rehabilitation principle of MIT [16]. Idiom is a unique Chinese word collocation, although its melody is slightly weak, but it has the characteristics of songs do not have: ① idiom collocation fixed and neat, composed of 4 words, has a good sense of rhythm; ② has a certain story background, conducive to the extraction of patient words and oral expression, and idiom story background characteristics, make the training more interesting; ③ has a wide public familiarity[16,17]. Inspired by MIT, the present study used these characteristics of Chinese idioms to train idiom reading for non-fluent aphasia patients in order to improve their language function.Computer as the main tool of information, with its fast, convenient, image, and other characteristics, in the world popularization.At the same time, with the development of the "Internet + medical" mode, computer plays a huge role in the medical field. Its multimedia changes, such as graphics, audio and animation, provide strong technical support for the rehabilitation of aphasia patients. Therefore, computer is considered as a useful means of language rehabilitation[18]. Computer-aided language evaluation for aphasia

Evaluation and computer-assisted aphasia rehabilitation treatment are two main aspects of computer application in aphasia rehabilitation care.Computer-assisted language assessment for aphasia refers to the conversion of a paper version of the aphasia assessment scale pictures and text on a computer and later presented by screen and speech, thus in the language assessment for patients with aphasia.Computer-assisted language function evaluation software for aphasia at home and abroad mainly includes: language disorder instrument ZM 2.1[19], Computer speech assessment and rehabilitation system TG PX 111[20]And the Parrot series for aphasia therapy[21]class.Computer-assisted aphasia rehabilitation care mainly consists of three main forms[22]: The first is to restore impaired language function through semantic, phonetic, or grammar characteristics, namely obstacle-oriented therapy; the second is to realize patients' daily life communication activities and train patients to use the residual language as effectively as possible; and the third is a participatory therapy by using computers to encourage more participation in social communication, in which most current aphasia rehabilitation computer software tend to be disorder -oriented therapy.Domestic common aphasia rehabilitation nursing computer software has language disorder instrument ZM2.1[19], Accessible computer language system U 1[23]And the computer speech evaluation and rehabilitation system TG PX 111[20]etc.; foreign computer software in computer script training Aphasia Scripts™[24], IMITATE computer-assisted aphasia treatment system[25]And the Parrot series of aphasia treatment software[21]class.According to Katz, the application of computers in aphasia rehabilitation is mainly divided

into two main categories: computer-assisted therapy (CAT) and computer-independent therapy (COT)[26]。

3.2 Study Purpose

Main purpose: To explore the rehabilitation effect of idiom training on non-fluent aphasia, in order to seek a new effective means of aphasia rehabilitation training to meet the rehabilitation needs of non-fluent aphasia patients.

3.3 Study design

This study is a randomized controlled study.No randomized or any study protocol-driven treatment will be administered or offered to the subjects during the course of the study.If clinically applicable, the treating physician should make the treatment decision and make the choice of treatment options.

.3.13 Subjects

The subject population in this study was patients with non-fluent aphasia after stroke.

Selection criteria:

(1) Was diagnosed with aphasia through the aphasia Screening Form of China Rehabilitation Research Center, and was a non-fluent aphasia patient;

(2) Primary school education or above;

(3) Clear awareness and cooperation, normal intelligence before the onset, and no history of mental illness;

(4) It is non-fluent aphasia after ischemic stroke, and the cerebral infarction site is the posterior inferior frontal gyrus of the dominant side, and the disease

In the stable period, you can adhere to sit alone for more than 30mi n;

(5) Informed and agreed to participate in this test, and sign the informed consent form;

(6) Age is from 45 to 70 years old

Exclusion criteria: (means the object that does not meet the study requirements even if they meet the inclusion criteria)

(1) Cognitive impairment;

(2) Mental disorders;

(3) Binocular corrected visual acuity <1.0 or visual field defect;

(4) Hearing impairment;

(5) Disarthria; loss of speech;

(6) Complete aphasia, Wernicke aphasia, transcortex sensory aphasia and transcortex mixed aphasia;

.23.3 Determine of sample size for study

Based on the method and purpose of this study, the pre-test was conducted, and the sample size was calculated using formula n = (+) 2 / [2 (sin-1 e-sin-1 c) 2]. Pe and Pc were the positive rates of the test group and the control, respectively (i. e., the proportion of patients with improved language function).

.33.3 Research methods and steps

As this is a randomized controlled study, the physician will decide on the treatment plan according to the instructions and the clinical pathway.The investigator will review the patient's medical history and test report and determine patient eligibility based on the inclusion and exclusion criteria.Patients must sign the latest IRB / IEC approved informed consent (I C F) before performing data collection.In this non-intervention study, the investigators collected clinical assessment data at baseline and after the intervention (2 weeks).

(1) Informed consent and enrollment

Subjects who provided informed consent and met all other inclusion / exclusion criteria were considered as enrolled in the study.

(2) Subject identification number

The numbers were grouped by random number table, and each subject was given a unique identification number (one researcher selected the second column in the third row according to the random number table, all numbers divided by 2, odd to the control group and even to the observation group).This identification number will be used for all study documents (such as a language assessment form, etc.).Furthermore, according to the data privacy regulations, unique identification numbers are allowed as long as it does not contain combined information that identifies the subject.

(3) Research process:

The observation group is receiving routine treatment (including medication, routine care in neurology department, and speech rehabilitation)

Teaching guidance, etc.) on the basis of computer assisted aphasia idiom reading training, the specific method for the use of computer software Powerpoint2003, computer screen in turn white background black idioms (idioms from the student practical idiom dictionary), a total of 5 0, the computer screen will display idioms, idioms in the form of words, idioms are equipped with auxiliary reading prompt material, including the corresponding idioms unsealed or expanded into the form of characters, words, sentences, paragraphs.At the bottom of the screen, there are the button of each prompt and the button to switch the next idiom. First, the speech rehabilitation teacher asks the patient to read the idiom. When the patient cannot read the idiom, click the corresponding prompt button (voice, video or picture prompt) to help the patient read.The training period is 2 weeks, per day For 40 minutes, the training ground is a clinical classroom.

The control group only underwent conventional treatment, including medication, routine care in neurology department, education and guidance of speech rehabilitation, but not regular speech rehabilitation training.Before and after the study, the Chinese aphasia examination method was used (ABC) to evaluate its language function.

(4) Data source / data collection process

Data from this study were obtained by extracting the routine clinical treatment records of the enrolled subjects.The investigator is required to fill in the study's e-case report form (eCRF) and or records based on the information entered in the patient medical records during the entire monitoring period.

(5) Data collection steps: (fill in by yourself according to the research content)

Baseline period: General information table and score of Aphasia Battery Of Chinese (ABC) scale and Comprehensive activities of daily living(CADL) scale

Routine diagnosis and treatment period: score of Aphasia Battery Of Chinese （ABC） scale and Comprehensive activities of daily living(CADL) scale

3.4 Data management

Subject data should be reported quickly, completely and correctly, and all steps related to data management should be recorded to check data quality and test implementation. To ensure the confidentiality of the database, it should have the maintenance and support procedures of the computer database. Design the clinical report form and the corresponding computer program that can be read and input by the computer. Subject assignment must be performed according to the randomized protocol determined by the trial design, and sealed codes for each study subject should be kept by the investigator.

System of original data records: 1) Before the start of the study, the project leader should discuss with the researchers and supervisors how to record the information about the clinical research in the original data and establish the requirements for the original records. 2) The format of the clinical research information record provided by the project leader in the original data (CRF table). 3) The original data is signed and dated by the completed researcher according to the industry practice of the medical documents. 4) The name of the study subject and the study number should be placed on each page of the data. 5) All corrections to the original data shall be made by

Correct investigator is signed and dated.6) All the information and data recorded on the case report form shall be recorded in the original data.7) Completion and handover of the case report form: CRF filling is made by the patient to find the corresponding entry serial number according to the patient case number and enter the CRF form. After the filling, the researchers will review the patients.8) The project informed consent was kept in each electronic CRF form after the patient.9) Fill in the completed case report form, after the intervention and early follow-up will be sent to the clinical trial data management personnel unified database and the project entry personnel double entry, kept by the researchers lock and save electronic original data, late follow-up data after the project designated entry personnel double entry.10) Data entry and modification: the relevant medical record information entered in each stage will be locked by the signature of the patient and the signature of the supervisor.All data will be entered by computer software.During this period, the questionable CRF form is forwarded to the investigator through the clinical supervisor for data review, and the investigator should answer and return as soon as possible.11) Data locking: the locked data file is not allowed to be changed again.The database will be submitted to the statistical analysts for statistical analysis as required in the statistical plan.

3.5 Statistical analysis

3.5.1 Statistical Methods

Statistical analysis was performed using the statistical software SPSS20.0, Data are represented in terms of ($x \pm s$), The study language function score and patient general data (age and disease duration) are the measurement data, Therefore, the t-test of two independent samples was used to compare between the experimental and

control groups, Two respective internal pre-posterior scores were compared using a paired sample t-test, Meverage of language function difference before and after the study using paired sample t-test; patient general data (including sex and stroke type) are count data, The chi-square test was used, Significance level =0.05.

3.5.2 Statistical software

SPSS20. 0

3.5.3 Research quality management

(1) The person in charge of this study has a high professional title, has presided over and completed a number of topics, published several papers related to the rehabilitation of elderly patients, and has the research basis for completing this study.

(2) The members of this study include Zhu Shanshan, chief physician of rehabilitation Department, and Lu Yinshan, chief physician of rehabilitation department. They have many years of experience in language rehabilitation training and can provide training tools and venues for research. In addition, the research composition

There are geriatric nurse Li Weiwei (graduate student) and two graduate students majoring in geriatric nursing, with experience and knowledge in geriatric rehabilitation, competent for research implementation.

3.6 Safety evaluation

The intervention of this study is language function rehabilitation training, without invasive operation and medication. During the intervention and during the intervention, the doctors doctors determine the suitability to conduct and continue to participate in language rehabilitation training.Any discomfort during the course of the intervention.Furthermore, the study interventions were performed in the patient's department without going out.

3.7 Expected study progress and completion date

September 13,2020-September 17,2021

Signature of the project leader:

April 10,2020
