# Supplementary material for: Effect of an extension speech training program based on Chinese idioms in patients with post-stroke non-fluent aphasia: A randomized controlled trial
Source: PLoS One. 2023 Feb 8;18(2):e0281335. doi: 10.1371/journal.pone.0281335 (PMC9907817; doi:10.1371/journal.pone.0281335)
Supplement: S3 Protocol — (PDF) [file pone.0281335.s005.pdf]

伦理审查同意函

伦审号	WDRY2020-K229	项目类型	科研
项目名称	计算机辅助非流畅性失语症成语朗读康复训练		
申办者	武汉大学人民医院（研究者自发）		
主要研究者	何小俊	承担学科	老年病科、神经内科、康复科
审查类别	☑初始审查 ☐复审 ☐跟踪审查		
审查方式	☐会议审查 ☐紧急会议审查 ☑快速审查		
审查文件	1. 科研项目伦理审查申请表<br>2. 非注册类临床研究项目受理表<br>3. 学术评议意见表<br>4. 非注册类临床研究立项申请表<br>5. 项目负责人简历<br>6. 研究方案（版本号：2.0；日期：2020-07-29）<br>7. 知情同意书（版本号：V2.0；日期：2020-07-29）<br>8. 一般资料信息表<br>9. 无经费资助声明（日期：2020-07-29）<br>10. 研究方案修正对比表<br>11. 研究方案（版本号：V3.0；日期：2020-11-15）<br>12. 知情同意书修正对比表<br>13. 知情同意书（版本号：V3.0；日期：2020-11-15）		

伦理委员会审评意见

同意进行临床研究

年度/定期跟踪审查频率	12 个月	截止日期	2021-12-11
主任委员/副主任委员签名	[signature]	日　　期	2020.12.11

武汉大学人民医院临床研究伦理委员会（盖章）

注意：（请仔细阅读）

1. 研究者应遵循伦理委员会批准的方案执行，实施过程应符合 NMPA/GCP 和赫尔辛基宣言的原则。
2. 在试验实施过程中，对研究方案和知情同意书等相关文件所作的任何修改，均需得到伦理委员会审查同意后方可实施。
3. 发生严重不良事件及可能影响风险受益比的任何事件和新信息须及时报告本院伦理委员会。
4. 接受伦理委员会持续审查的项目，请在到期前 1 个月（无论试验开始与否）提出再次审查的申请。
5. 如有违背/偏离方案或暂停/提前终止的试验项目，应及时以书面文件报告本院伦理委员会；临床试验结束后，须及时向伦理委员会提交结题报告。
6. 同意函有效期 1 年（自批准之日起），如试验逾期未实施即自行废止。
