# Supplementary material for: Effect of an extension speech training program based on Chinese idioms in patients with post-stroke non-fluent aphasia: A randomized controlled trial
Source: PLoS One. 2023 Feb 8;18(2):e0281335. doi: 10.1371/journal.pone.0281335 (PMC9907817; doi:10.1371/journal.pone.0281335)
Supplement: S4 Protocol — (PDF) [file pone.0281335.s006.pdf]

AF SOP/3.17-02/6.0

Ethical review and approval letter

Ethics number	WDRY2020-K229	Project type	scientific research
project name	Computer-assisted non-fluent aphasia idioms rehabilitation training		
The applicant	People's Hospital of Wuhan University (spontaneous)		
Principal investigator	He Xiaojun	Department	Department of Neurology, Rehabilitation and Geriatrics
Review category	Initial review ☐ reexamine ☐ To track the review ☐		
Review method	Day meeting examination review ☐ Emergency Meeting ☐ Review Quick☐		
review file	1. Application form for ethical review of scientific research projects<br>2. Acceptance form for non-registered clinical research items<br>3. Academic comment and opinion form<br>4. Application form for non-registered clinical research project approval<br>5. Project Leader Resume<br>6. Study protocol (version No.: 2.0; Date: 2020.0729)<br>7. Informed Consent (Version Number: V2.0; Date: 2020.07.29)<br>8. General Information Information Table<br>9. No Funding Statement (Date: 2020-07-29)<br>10. Revised comparison table of the study protocol<br>11. Study protocol (version No.: V3.0; Date: 2020-11-15)<br>12. Revised ison table table the informed consent form<br>13. Informed Consent (Version No: V3.0; Date: 11.15,2020)		

Review opinions of the Committee			
Agree to conduct clinical trials<br>All eight from X Letter male points			
Annual / regular follow-up review frequency	12 Months	Deadline	2021-12-11
Signature of ethics committee director/Deputy director		date	2020-12-11
r Ethics Committee of Clinical Research, People's Hospital of Wuhan University			

Attention (Please read carefully)

1 The researcher shall follow the implementation of the erC protocol, and the implementation process shall comply with the principles of NMPA/GCP and Helsinki Declaration

2, Any modifications to the study protocol and informed consent documents during the trial implementation are required to be reviewed by the ethics committee

3, The occurrence of serious adverse events and any events and new information that may affect the risk-benefit ratio shall be timely reported to the ethics committee of the hospital.

4, Accept the ethics committee continued to review the item, jing before the expiration of [months (whether the trial began or not) before the review!blue or green.

5, If J has any violation / high scheme or suspension / early trial termination, it shall report to the ethics committee of the hospital in a written document;after the completion of the clinical trial.

6, The consent letter is valid for [year (from the date of approval), and shall be abolished by itself if the test is not implemented within the time limit.

Add: 99 Zhangzhidong Road, Wuchang District, Wuhan 430060 Tel: 027-88041911-81353
