# Supplementary material for: Effect of an extension speech training program based on Chinese idioms in patients with post-stroke non-fluent aphasia: A randomized controlled trial
Source: PLoS One. 2023 Feb 8;18(2):e0281335. doi: 10.1371/journal.pone.0281335 (PMC9907817; doi:10.1371/journal.pone.0281335)
Supplement: S1 Fig — (PDF) [file pone.0281335.s007.pdf]

- Read：“愚公移山”（Mr. Yu Removed the Mountain）
- Q&A：Can you tell me what idiom is in the picture？

…….

Word

Read：
“愚”/yu/
“公”/gong/
“移”/yi/
“山”/shan/

Character

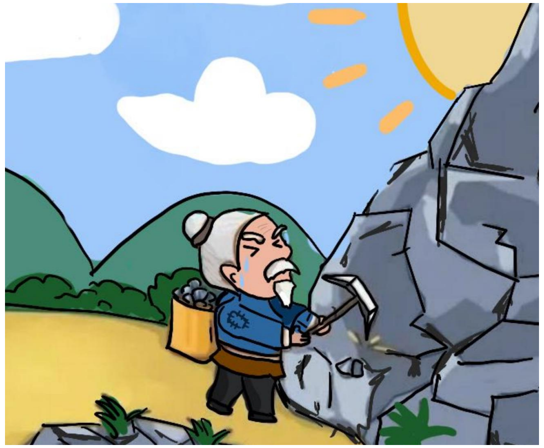

Sentence

- Read: “我们要像愚公移山般坚持”（Stick to it like Mr. Yu moving mountains）
- Q&A：What kind of person is the main character？

…….

Paragraph

- Read：（The text of this idiom story）
- Q&A：Please describe the story in the picture to me as much as possible？

……
