# Supplementary material for: Effect of an extension speech training program based on Chinese idioms in patients with post-stroke non-fluent aphasia: A randomized controlled trial
Source: PLoS One. 2023 Feb 8;18(2):e0281335. doi: 10.1371/journal.pone.0281335 (PMC9907817; doi:10.1371/journal.pone.0281335)
Supplement: Attachment — Submitted filename: Response to Reviewers.docx [file pone.0281335.s008.docx]

*We sincerely thank the editor and all reviewers for their valuable feedback that we have used to improve the quality of our manuscript. The reviewer comments are laid out below in italicized font and specific concerns have been numbered. Our response is given in normal font and changes/additions to the manuscript are given in the* blue (name：Super pei)*text.*

***Question 1****：I am also not sure why they would have conducted a per-protocol, rather than an intent-to-treat, analysis. What was their reasoning for this?*

In three of the 70 patients we enrolled, the outcome variables were missing because of failure to complete the intervention and outcome evaluation during the course of the trial for reasons superior to deterioration, cognitive decline, and voluntary withdrawal. Since the missing outcome variable was a continuous variable, we did not find a very satisfactory complement method for missing data, and the missing data were very few (less than 5%), so we chose to analyze only non-missing data (*per-protocol* analysis).We speculate that the bias from data deletion (PP analysis) may not necessarily be greater than that from data complement (ITT analysis).

Under the reminder of the reviewer's opinion, we also used *intent-to-treat analysis* for the data again, and the missing data were filled in with baseline measurement data. The analysis showed that there was no significant difference between the PP analysis and ITT analysis. In the manuscript, we also add the results of the ITT analysis.The specific results of PP analysis and ITT analysis are as follows:

|  |  | Per-protocol Analysist | | | Intent-to-treat Analysis | | |
| --- | --- | --- | --- | --- | --- | --- | --- |
|  |  | Group I  n=33 | Group II n=34 | P-value | Group I  n=34 | Group II n=36 | P-value |
| Gender (n) | |  |  | 0.63 |  |  | 0.7 |
|  | Female | 8 | 10 |  | 9 | 11 |  |
|  | Male | 25 | 24 |  | 25 | 25 |  |
| Age (Mean (SD)) | | 65.4(10.6) | 64.7(12.2) | 0.79 | 65（10.7） | 64.7（13.3） | 0.92 |
| Education level (n) | |  |  | 0.93 |  |  | 0.8 |
|  | Primary school | 4 | 6 |  | 4 | 7 |  |
|  | Junior high school | 14 | 14 |  | 14 | 15 |  |
|  | High school | 11 | 10 |  | 12 | 10 |  |
|  | University degree or above | 4 | 4 |  | 4 | 4 |  |
| Stroke type (n) | |  |  | 0.56 |  | | 0.63 |
|  | infarction | 28 | 27 |  | 28 | 28 |  |
|  | hemorrhage | 5 | 7 |  | 6 | 8 |  |
| Aphasia type (n) | |  |  | 0.37 |  | | 0.6 |
|  | BA | 21 | 18 |  | 21 | 20 |  |
|  | TCM | 12 | 16 |  | 13 | 16 |  |
| Hemiplegia side (n) | |  |  | 0.84 |  | | 0.93 |
|  | Left | 4 | 6 |  | 5 | 6 |  |
|  | Right | 20 | 18 |  | 20 | 19 |  |
|  | Bilateral | 1 | 2 |  | 1 | 2 |  |
|  | None | 8 | 8 |  | 8 | 9 |  |
| Time（days）(Median（interquartile range) ) | | 15(1) | 14.5(1) | 0.84 | 15（1） | 14（1） | 0.78 |

|  |  | Per-protocol Analysist | | | Intent-to-treat Analysis | | |
| --- | --- | --- | --- | --- | --- | --- | --- |
|  |  | Group I  n=33 | Group II n=34 | P-value | Group I  n=34 | Group II n=36 | P-value |
| ABC score (Mean (SD) ) | |  |  |  |  |  |  |
| Expressing | Before the intervention | 54.5（22.3） | 53.1(24.5) | 0.816 | 55.8(21.7) | 52.4(25.9) | 0.555 |
|  | After the intervention | 82.3（13.7） | 73.2（20.7） | 0.038* | 82.6（13.3） | 71.5（22.6） | 0.016* |
|  | P-value | 0** | 0** |  | 0** | 0** |  |
| Comprehension | Before the intervention | 77.4(8.0) | 76.6(9.1) | 0.733 | 77.8(7.3) | 75.9(10.2) | 0.395 |
|  | After the intervention | 87.5（9.8） | 82（11） | 0.037* | 87.9（9.6） | 80.7（11.5） | 0.006** |
|  | P-value | 0** | 0** |  | 0** | 0** |  |
| Reading | Before the intervention | 55.3(19.6) | 58.1(23.3) | 0.586 | 55.8(19.0) | 57(24.2) | 0.822 |
|  | After the intervention | 80.5（15.4） | 69.7（22.9） | 0.028* | 81（15） | 67.3（23.78） | 0.006** |
|  | P-value | 0** | 0** |  | 0** | 0** |  |
| Writing | Before the intervention | 77.7(14.0) | 79.1(17.3) | 0.825 | 78(13.7) | 75.9(22.2) | 0.881 |
|  | After the intervention | 83.1（15.4） | 84.7（9.6） | 0.752 | 81.9（15.4） | 80.7（18.7） | 0.858 |
|  | P-value | 0.051 | 0.124 |  | 0.051 | 0.123 |  |
| CADL score (Mean (SD) ) | |  |  |  |  |  |  |
|  | Before the intervention | 68.6(34.6) | 71.97(37.7) | 0.708 | 70.8(33.3) | 70.7(38.4) | 0.986 |
|  | After the intervention | 110.67（25.4） | 95.8（32.8） | 0.043* | 110.4（25） | 94.3（33.3） | 0.026* |
|  | P-value | 0** | 0** |  | 0** | 0** |  |

***Question 2****：The objectives/hypothesis, although referenced, are not explicitly stated: (e.g., “We hypothesize that the idiom based speech training program will be more effective at xxxxx , as demonstrated by increased scores on xxx, than the conventional speech training program.”)*

Thank you very much for your reminding.In the manuscript we added more explicitly hypotheses

***Question 3****：The authors state that the study was powered for an effect size=0.8. Could they please indicate what this means in terms of score improvement, or difference in expected scores between the two groups?*

Effect Size (ES) is an index to measure the strength of association between independent variables and dependent variables, that is, to reflect the Size of statistical test Effect or treatment Effect. The effect size index we take here is Cohen's d, which reflects the difference between two populations under the influence of something, and is a commonly used effect size index for t-test. We calculated the experimental data of the previous similar studies (Zhou J(2021), Zhu XJ(2021), Li WW(2019)), and their effect sizes Cohen's d were all greater than 0.8. Therefore, we considered this kind of study as a large-effect index test. Therefore, according to the criteria proposed by Cohen(1992), In this study, the effect size was 0.8 when the sample size was calculated.

***Question 4****：p.6. Were only patients with a first stroke included in the trial, or could patients with a history of stroke be enrolled?*

In order to avoid confounding factors caused by multiple strokes and improve the homogeneity of the sample, patients with first stroke were included in our study.We have corrected the expression of this aspect in the inclusion and exclusion criteria of the submitted manuscript

***Question 7****：The authors state that both parametric and non-parametric tests were used to compare samples as appropriate. Table 1 should reference which tests were used for each comparison, and any non-normal data should be summarized using the median and interquartile range (some of the test scores do not appear to be normally distributed).*

Thanks for your suggestion. We have modified the incorrect expression in Table 1 and added an explanation of the data tests in Table 1 Legend

***Question 8****：The authors report scores of the two groups after rehabilitation treatment, but in fact, the correct comparison is between the before-and-after differences between the two groups. Therefore, the pre- and post-differences, rather than the “post” scores for each, should be reported. From the description of statistical tests used, it appears that the authors analyzed the data correctly (since both paired and unpaired tests were used). A table which clearly shows the within and between group differences would be useful to clarify this (Group 1, differences between pre- and post-intervention measurements, the same for Group 2, and the comparison between Group 1 and Group 2).*

We originally described the initial evaluation results and whether there were differences between groups in **Table 1**, but we found that it was not conducive to readers' reading after the reviewer's suggestion.In the manuscript submitted this time, the initial evaluation results in our baseline data form were moved to the parts of Language Function Evaluation and Daily Communication Skill respectively.

In addition, in order to present more specific data information, we changed the presentation method of the results from the statistical graph (original version **Fig 3, Fig 4)** to the statistical table (**Table 2, Table 3)**

***Question 9****：Did the authors consider doing a repeated measures analysis so that they could adjust for co-morbidities, prior strokes (if allowed), age, etc.?*

Thank you for this suggestion. It would have been interesting to explore this aspect. However, in our study, this would be difficult to implement because we unfortunately only counted two outcome variables (pre-intervention and post-intervention)

***Question 10****：Please write out BA and TCM before using abbreviations.*

We were really sorry for our careless mistakes. Thank you for your reminder.We have added the full name in the manuscript submitted this time

***Question 11****：Again, it is not clear to me why a per protocol analysis was done rather than an intent-to-treat analysis. The authors need to explain and justify their reason for so doing.*

We have already answered this question in **Question 1**. we are not sure if it would be appropriate to list both PP analysis results and ITT analysis results (it would make the table less concise). we would be very grateful if you could give us some suggestions.

***Question 12****：The first paragraph of the Discussion belongs in the Introduction and should be incorporated there (some of it is redundant). The Discussion section is the place to discuss the meaning of the results, not outline the reasons for the study itself.*

Thank you for pointing this out. According to the reviewer's opinion, we changed the relevant paragraph to the introduction section and deleted the duplicate part.

***Question 13****：Second paragraph: I believe that the authors mean that the CHANGE in scores was higher in Group 1 than in Group 2. This should be clarified throughout the paper. It is never clear what the percentage changes in the two groups were, or how big of a difference there was between them, since only post-intervention scores are reported.*

We have added the suggested content to the manuscript on **Table 2** and **Table 3** of the results section

***Question 14****：Fourth paragraph. The second sentence has the following note: “[Error! Reference source not found.” This implies that that the paper was not carefully proofread before submission. The actual references for these “studies” need to be added.*

We were really sorry for our careless mistakes. Thank you for your reminder.We have added references in manuscript

***Question 15****：Fifth paragraph: The authors should refrain from using the words “we…proved…” Their study supports an association between their method and improved language skills after stroke, but does not prove it.*

Thanks for your careful checks. We are sorry for our carelessness. Based on your comments, we have made the corrections to the description of the whole manuscript more appropriate

**In addition**

We tried our best to improve the manuscript and made some changes and language editing to the manuscript. These changes will not influence the content and framework of the paper. And here we did not list the changes but marked in red( name:Author) in the revised paper. We appreciate for Editors/Reviewers’ warm work earnestly and hope that the correction will meet with approval.
