# Supplementary material for: Effect of an extension speech training program based on Chinese idioms in patients with post-stroke non-fluent aphasia: A randomized controlled trial
Source: PLoS One. 2023 Feb 8;18(2):e0281335. doi: 10.1371/journal.pone.0281335 (PMC9907817; doi:10.1371/journal.pone.0281335)
Supplement: Attachment — Submitted filename: Response to Reviewers.docx [file pone.0281335.s009.docx]

We sincerely thank the editor and all reviewers for their valuable feedback that we have used to improve the quality of our manuscript. .The reviewer comments are laid out below in italicized font and specific concerns have been numbered. Our response is given in normal font and changes/additions to the manuscript are given in the blue (name：Super pei)text. According to the reviewer’s comments, we have revised the manuscript extensively. If there are any other modifications we could make, we would like very much to modify them and we really appreciate your help. We hope that our manuscript could be considered for publication in your journal. Thank you very much for your help

1. *Please do not use point bullets for the eligibility of your subjects.*

Thank you very much for your advice. We have removed the point bullets in the Inclusion criteria and Exclusion criteria and changed them to such formats as (a)(b)(c), etc

2. *Why do you use either PP and intention to treat rather than only ITT analysis？*

We thank the reviewer of pointing out this issue. According to the suggestions of reviewers and editors, we have changed the statistical analysis method.We updated the manuscript to intent-to-treat analysis for the data , and the missing data were filled in with baseline measurement data.

3. *Do the authors make an adjustment of the other confounding factors ? education level, side of weakness, type of treatment ?*

Thank you very much for your question，In order to avoid confounding bias as much as possible, we did the following methods. Firstly, in the recruitment phase of the experiment, we strictly followed the random allocation principle to ensure that potential confounding factors could be evenly distributed between the experimental group and the control group. Second, in the analysis stage of experimental data, we compared confounding factors such as education level, age, stroke type and aphasia type in the intervention group and the control group in the baseline data. The results showed that the p values of these factors were all greater than 0.05, and the baseline data were comparable. In addition, inspired by the comments of editors and reviewers, We conducted stratified analysis according to stroke type, education level, aphasia type and hemiplegic side.The results are shown below. The intra-group differences in the results of stratification analysis have no significant conflict with the original results.However, for post-intervention intergroup analyses, stratified analyses reduced their effect size.We have the following explanation for this change.First and foremost, since our overall sample size was only 70,in the stratified analysis, our sample size was significantly reduced, which was closely related to the change of effect size. Secondly, compared with the difference between groups before the intervention, the P-value after the intervention has significantly increased. After intervention, the mean scores of the intervention group were higher than those of the control group.In addition, we noted that even though the p values between the groups after our intervention did not reach statistical significance, most of the p values were at a low level.

| Stratified analysis of aphasia types | | P-values for intra-group comparisons | P-values for intra-group comparisons | P-values for comparison between groups before intervention | P-values of post-intervention comparison between groups |
| --- | --- | --- | --- | --- | --- |
| TCM | | Group1 n=13 | Group2n=16 |  |  |
|  | Expressing | 0* | 0* | 0.803 | 0.116 |
|  | Comprehension | 0* | 0* | 0.916 | 0.034* |
|  | Reading | 0* | 0.006* | 0.424 | 0.11 |
|  | Writing | 0.014*（n=8） | 0.263（n=7） | 0.303 | 0.878 |
|  | CADL | 0* | 0.013* | 0.628 | 0.017* |
| BA | | Group1 n=21 | Group2n=20 |  |  |
|  | Expressing | 0* | 0* | 0.4 | 0.023* |
|  | Comprehension | 0.002* | 0.05* | 0.243 | 0.022* |
|  | Reading | 0* | 0* | 0.592 | 0.004* |
|  | Writing | 0.732（n=5） | 0.023*（n=8） | 0.373 | 0.797 |
|  | CADL | 0* | 0.0* | 0.999 | 0.152 |
| Stratified analysis of stroke types | | P-values for intra-group comparisons | P-values for intra-group comparisons | P-values for comparison between groups before intervention | P-values of post-intervention comparison between groups |
| Cerebral hemorrhage | | Group1 n=6 | Group2n=8 |  |  |
|  | Expressing | 0.024* | 0.003* | 0.816 | 0.122 |
|  | Comprehension | 0.053* | 0.033* | 0.943 | 0.29 |
|  | Reading | 0.028* | 0.209 | 0.521 | 0.179 |
|  | Writing | 0.766（n=4） | 0.291（n=3） | 0.505 | 0.863 |
|  | CADL | 0.024* | 0.03* | 0.498 | 0.064* |
| Cerebral infarction | | Group1 n=28 | Group2n=28 |  |  |
|  | Expressing | 0* | 0* | 0.927 | 0.309 |
|  | Comprehension | 0* | 0.002* | 0.459 | 0.014* |
|  | Reading | 0* | 0* | 0.631 | 0.185 |
|  | Writing | 0.042*（n=9） | 0.292（n=12） | 0.427 | 0.334 |
|  | CADL | 0* | 0* | 0.988 | 0.097* |
| Stratified analysis of hemiplegia types | | P-values for intra-group comparisons | P-values for intra-group comparisons | P-values for comparison between groups before intervention | P-values of post-intervention comparison between groups |
| Bilateral hemiplegia was removed | | Group1 n=33 | Group2n=34 |  |  |
|  | Expressing | 0* | 0* | 0.862 | 0.05* |
|  | Comprehension | 0* | 0* | 0.467 | 0.024* |
|  | Reading | 0 | 0* | 0.492 | 0.021* |
|  | Writing | 0.051*（n=13） | 0.124（n=15） | 0.804 | 0.187 |
|  | CADL | 0* | 0* | 0.761 | 0.813 |
| All types of hemiplegia | | Group1 n=26 | Group2n=27 |  |  |
|  | Expressing | 0* | 0* | 0.846 | 0.154 |
|  | Comprehension | 0* | 0* | 0.826 | 0.015* |
|  | Reading | 0* | 0* | 0.846 | 0.016* |
|  | Writing | 0.08*（n=5） | 0.12（n=5） | 0.49 | 0.541 |
|  | CADL | 0* | 0* | 0.591 | 0.067* |
| Right hemiplegia was removed | | Group1 n=14 | Group2n=17 |  |  |
|  | Expressing | 0* | 0* | 0.509 | 0.041 |
|  | Comprehension | 0* | 0.015* | 0.709 | 0.077* |
|  | Reading | 0.001* | 0.003* | 0.497 | 0.247 |
|  | Writing | 0.051*（n=13） | 0.124（n=15） | 0.804 | 0.813 |
|  | CADL | 0* | 0* | 0.539 | 0.102 |
| Left hemiplegia was removed | | Group1 n=29 | Group2n=30 |  |  |
|  | Expressing | 0* | 0* | 0.288 | 0.005* |
|  | Comprehension | 0* | 0.002* | 0.208 | 0.003* |
|  | Reading | 0* | 0* | 0.782 | 0.001* |
|  | Writing | 0.046*（n=8） | 0.058*（n=9） | 0.399 | 0.514 |
|  | CADL | 0* | 0* | 0.652 | 0.031* |
| Stratified analysis of educational types | | P-values for intra-group comparisons | P-values for intra-group comparisons | P-values for comparison between groups before intervention | P-values of post-intervention comparison between groups |
| Elementary and junior high school education | | Group1 n=18 | Group2n=22 |  |  |
|  | Expressing | 0* | 0* | 0.615 | 0.037* |
|  | Comprehension | 0* | 0* | 0.709 | 0.02* |
|  | Reading | 0* | 0* | 0.803 | 0.06* |
|  | Writing | 0.08*（n=9） | 0.12（n=9） | 0.135 | 0.187 |
|  | CADL | 0* | 0* | 0.953 | 0.14 |
| High school and college education | | Group1 n=16 | Group2n=14 |  |  |
|  | Expressing | 0* | 0* | 0.927 | 0.209 |
|  | Comprehension | 0.001* | 0* | 0.459 | 0.214 |
|  | Reading | 0* | 0* | 0.631 | 0.185 |
|  | Writing | 0.205（n=4） | 0.539（n=9） | 0.427 | 0.334 |
|  | CADL | 0* | 0.003* | 0.988 | 0.117 |
