# Supplementary material for: Effect of an extension speech training program based on Chinese idioms in patients with post-stroke non-fluent aphasia: A randomized controlled trial
Source: PLoS One. 2023 Feb 8;18(2):e0281335. doi: 10.1371/journal.pone.0281335 (PMC9907817; doi:10.1371/journal.pone.0281335)
Supplement: Attachment — Submitted filename: Response to Reviewers.docx [file pone.0281335.s010.docx]

We sincerely thank the editor and all reviewers for their valuable feedback that we have used to improve the quality of our manuscript. .The reviewer comments are laid out below in italicized font and specific concerns have been numbered. Our response is given in normal font and changes/additions to the manuscript are given in the red text. According to the reviewer’s comments, we have revised the manuscript extensively. If there are any other modifications we could make, we would like very much to modify them and we really appreciate your help. We hope that our manuscript could be considered for publication in your journal. Thank you very much for your help!

1. *Please begin the discussion with the meaning of the results, not outline the reasons for the study itself.*

Thank you very much for your suggestion. We have revised the beginning of the discussion section,and the outline of the study at the beginning of the discussion was deleted.

2. *If you do intention to treat analysis, please begin with the flowchart.*

Thank you very much for your prompt, we decided to use ITT analysis, and under your reminder, we have revised relevant parts of the paper, including the summary, description of data analysis, flow chart, and result part.

***3****.Please remove the bullet for the intervention section.*

We are sorry that we made a mistake in the format, we have removed the bullet for the intervention section

***4****.Please complete the p value, in the intra group analysis.*

Thank you very much for your suggestion. In Table 2 and Table 3, we have added more accurate p values（Change from p value =0** to p value< 0.001*)

***5.****Second paragraph: I believe that the authors mean that the CHANGE in scores was higher in Group 1 than in Group 2. This should be clarified throughout the paper. It is never clear what the percentage changes in the two groups were, or how big of a difference there was between them, since only post-intervention scores are reported.*

We are really sorry that we didn't explain it clearly here due to our inappropriate language. Under your suggestion, We have supplemented pre-intervention scores of the group I and the group II in Table 2 and Table 3. Meanwhile, we also added the percentage increase of the scores of the two groups in the first paragraph of the discussion.We would like to thank you for your suggestions to make the description of our results clearer.

We hope that the changes we’ve made resolve all your concerns about the article. We are more than happy to make any further changes that will improve the paper and/or facilitate successful publication.Thanks very much for your attention to our paprer.
